# Genome-Wide Characterization and Expression Analysis of CsPALs in Cucumber (*Cucumis sativus* L.) Reveal Their Potential Roles in Abiotic Stress and Aphid Stress Tolerance

**DOI:** 10.3390/plants13182537

**Published:** 2024-09-10

**Authors:** Jieni Gu, Hamza Sohail, Lei Qiu, Chaoyan Chen, Haoyu Yue, Ziyi Li, Xiaodong Yang, Lili Zhang

**Affiliations:** College of Horticulture and Landscape Architecture, Yangzhou University, Yangzhou 225009, China; 211803103@stu.yzu.edu.cn (J.G.); hamzasohail@yzu.edu.cn (H.S.); 030824ql@gmail.com (L.Q.); mz120221435@stu.yzu.edu.cn (C.C.); 233701167@stu.yzu.edu.cn (H.Y.); 221803308@stu.yzu.edu.cn (Z.L.); yxd@yzu.edu.cn (X.Y.)

**Keywords:** salinity, drought, PAL, aphids, genome wide, cucumber

## Abstract

Phenylalanine ammonia lyase (PAL) is a pivotal enzyme in the phenylalanine metabolic pathway in plants and has a crucial role in the plant’s response to environmental stress. Although the PAL family has been widely studied in many plant species, limited is known about its particular role in cucumbers under stress. We investigated the physicochemical properties, gene structure, gene duplication events, conserved motifs, cis-acting elements, protein interaction networks, stress-related transcriptome data, and quantitatively validated key stress-related genes. The main results indicated that 15 *PAL* genes were grouped into four clades: I, II, and III when arranged in a phylogenetic tree of *PAL* genes in angiosperms. The analysis of the promoter sequence revealed the presence of multiple cis-acting elements related to hormones and stress responses in the cucumber *PAL* genes (*CsPALs*). The analysis of protein interaction networks suggested that CsPAL1 interacts with eight other members of the PAL family through CsELI5 and CsHISNA, and directly interacts with multiple proteins in the 4CL family. Further investigation into the expression patterns of *CsPAL* genes in different tissues and under various stress treatments (NaCl, Cu^2+^, Zn^2+^, PEG6000, aphids) demonstrated significant differential expression of *CsPALs* across cucumber tissues. In summary, our characterization of the *CsPAL* family offers valuable insights and provides important clues regarding the molecular mechanisms of *CsPALs* in managing abiotic and biotic stress interactions in cucumbers.

## 1. Introduction

Phenylalanine ammonia lyase (PAL) initiates the phenylpropanoid pathway. It acts as a catalyst for the conversion of L-phenylalanine, which is produced by the shikimate pathway, into trans-cinnamic acid [1]. This enzyme plays a crucial role in bridging primary and secondary metabolism in plants, as it provides the necessary building blocks for different branches of phenylpropanoid metabolism. The pathway produces intermediate compounds including trans-cinnamic acid, coumaric acid, ferulic acid, and salicylic acid, which are further converted into coumarins, chlorogenic acid, or CoA esters, ultimately resulting in the formation of lignin, flavonoids, and other secondary metabolites. These chemicals play a role in several plant biological processes, including morphogenesis, growth and development, resistance to pathogen invasion, and responses to environmental stimuli [2,3,4]. Alterations in PAL activity have a strong correlation with plant resistance to diseases and the ability to withstand stress, making it a reliable indicator for environmental and biological stressors.

*PAL* genes were first obtained from barley (*Hordeum vulgare*) [5], and subsequent genome sequencing has revealed that the model plant *Arabidopsis thaliana* contains four unique *PAL* genes [6,7,8,9,10], specifically *AtPAL1*, *AtPAL2*, *AtPAL3*, and *AtPAL4* [9]. However, only *AtPAL1* and *AtPAL2* have been thoroughly studied in terms of their functions [8]. Expression analysis demonstrates that expression levels of *PAL* genes vary under different conditions and are frequently spatiotemporal and tissue-specific [11]. Therefore, it is crucial to analyze and investigate each *PAL* gene from a variety of perspectives to enhance plant stress resistance.

*PAL* genes significantly enhance plant tolerance and resistance under various abiotic and biotic stress conditions by regulating the synthesis of secondary metabolites and hormones. *PAL* genes are triggered in response to abiotic stressors such as heavy metals, high salt levels, drought conditions, cold temperatures, and waterlogging. The tolerance to drought stress is greatly enhanced in *pal1 pal2* double mutants in *Arabidopsis* [12]. The accumulation of suberin polyphenolics (SPP) and suberin polyaliphatics (SPA) was facilitated by the upregulation of *PAL* genes in potatoes under Ca^2+^ treatment, which contributed to the plant’s resistance to heavy metal stress [13]. Cold priming primarily stimulates the synthesis of salicylic acid (SA) through the PAL pathway, and when wheat is exposed to low temperatures, the *PAL* genes and endogenous SA levels are greatly increased [14]. Under waterlogging stress, wheat’s *PAL* gene expression is increased, raising SA concentration, triggering aerenchyma formation, and stimulating the production of adventitious roots, allowing wheat plants to react to waterlogging through morphological alterations [15]. In terms of biotic stress tolerance, the *PAL* gene enhances plant resistance by regulating the production of secondary metabolites and hormone levels. In maize, *PAL* genes confer resistance to sugarcane mosaic virus by promoting the accumulation of SA [16]. Increased expression of *OsPAL8* in rice stimulates the production and accumulation of SA and lignin, greatly improving rice’s ability to withstand brown planthopper infestation [17]. Furthermore, aphid infestation in susceptible wheat varieties leads to a significant increase in the expression of *PAL* genes. This is mainly caused by the aphid’s probing, which triggers hormone-dependent responses including SA and jasmonic acid (JA) [18].

Cucumber (*Cucumis sativus L.*) is an important vegetable crop that is widely cultivated globally and is highly valued for its nutritional value and economic significance [19]. Cucumbers are rich in vitamins and minerals, making them a major component of many diets [20]. Additionally, this crop serves as an important model system for studying plant sex determination and vascular tissue formation [21,22]. The growth and development of cucumbers are susceptible to various adverse stresses, such as heavy metals [23], waterlogging [24], dehydration [25], heat stress [26], and aphids [27], which can lead to reduced yield and quality. Utilizing biotechnological breeding techniques is an important approach to improve crop yield, quality, and stress resistance. This study aims to comprehensively explore the function of the *PAL* genes in cucumber in response to abiotic and aphid-induced stress by identifying and analyzing their expression profiles. The findings are intended to provide theoretical support for the green and efficient production of cucumber.

## 2. Results

### 2.1. Identification of PAL Genes in Cucumber

To identify *PAL* genes in cucumber, a Blastp and HMM search was performed on the Cucumber Chinese Long V3 genome using four *AtPAL* protein sequences [28]. Through this process, 15 *CsPAL* genes with PAL domains were successfully identified and named *CsPAL1* to *CsPAL15* based on their chromosomal positions. The open reading frame lengths of the PAL family ranged from 927 to 2154 bp, corresponding to protein lengths of 308 to 717 amino acids. Except for *CsPAL3*, the isoelectric points (PI) of the other *CsPAL* proteins ranged from 5.33 to 6.37. Additionally, except for *CsPAL2* and *CsPAL3*, the molecular weights of the other *CsPAL* proteins ranged from 77.17 to 78.37 KDa (Table S1).

**Figure 1:**
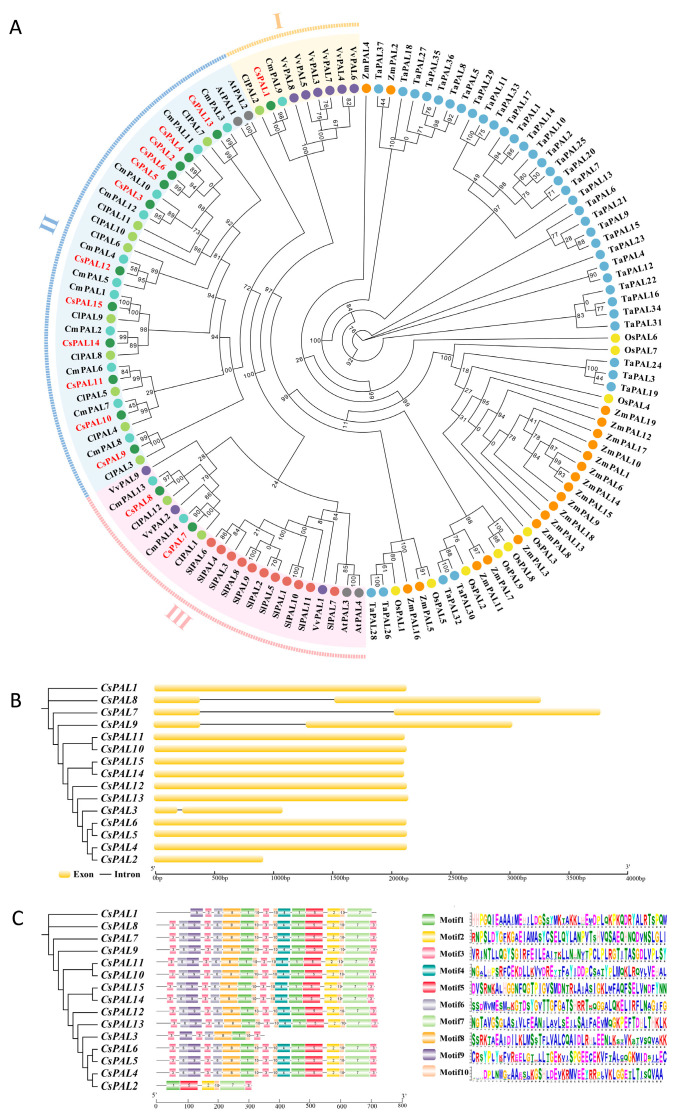
Phylogenetic tree, gene structure, and motif patterns of CsPAL proteins, and their chromosomal locations. (**A**) Phylogenetic tree constructed using the full-length sequences of CsPAL proteins, with each node supported by 1000 replicates. Different colored circular markers: Gray: *Arabidopsis thaliana*; Dark Green: *Cucumis sativus*; Light Green: *Citrullus lanatus*; Cyan: *Cucumis melo*; Purple: *Vitis vinifera*; Red: *Solanum lycopersicum*; Yellow: *Oryza sativa*; Orange: *Triticum aestivum*; and Blue: *Zea mays*. (**B**) Yellow rounded rectangles and black lines represent CDS (coding sequences or exons) and introns in cucumber, respectively. (**C**) Amino acid motifs (numbered 1–10) in CsPAL proteins are displayed in ten colored boxes, with black lines indicating amino acid lengths. For motif logos, the relative size of each letter represents its frequency of occurrence in the sequence. The height of each letter is proportional to the frequency of the corresponding base at that position.

**Figure 2:**
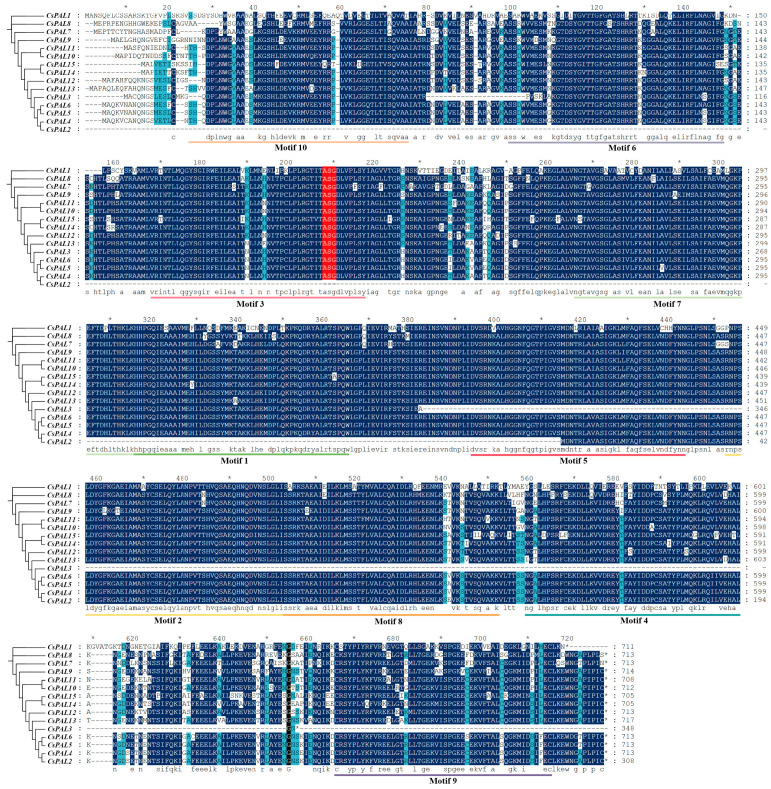
Multiple sequence alignment of cucumber PAL proteins. The red part is ASG, the active center of CsPAL. Conserved percent: Black = 100%; Dark blue ≥ 75%; Light blue ≥ 50%. Different colored lines represent different motifs 1–10. The asterisk (*) at the end of a protein sequence represents the stop codon.

To study the evolutionary relationships within the cucumber PAL gene family, a phylogenetic tree was constructed among cucumber, Arabidopsis, tomato, melon, watermelon, grape, corn, wheat, and rice (Figure 1A). According to the classification results from the phylogenetic tree, *CsPAL* genes can be divided into three subfamilies: one gene in subfamily I, twelve genes in subfamily II, and two genes in subfamily III. The PAL family proteins of monocotyledonous and dicotyledonous plants show clear boundaries in the clustering of the evolutionary tree, suggesting that during plant evolution, PAL proteins in monocots and dicots have evolved in relatively independent directions based on their actual needs for growth, development, or stress response. To better understand the gene evolutionary process, the exon-intron structure of the cucumber *PAL* genes was further analyzed. The analysis showed that the number of exons in *CsPAL* genes ranged from one to two, indicating high conservation (Figure 1B). The conserved motifs were analyzed using the MEME online tool, identifying 10 different motifs (named motifs 1–10). Except for *CsPAL2* and *CsPAL3*, *CsPAL* genes contained motifs 1–10, with *CsPAL4*–*CsPAL15* showing the same motif distribution, demonstrating conservation and similar functions. *CsPAL2* contained only motifs 1, 2, 3, 5, and 7, while *CsPAL3* contained motifs 3, 6, 8, 9, and 10 (Figure 1C). Multiple sequence alignment results (Figure 2) demonstrated that, except for *CsPAL2* and *CsPAL3*, most *CsPAL* domains are highly conserved.

### 2.2. Analysis of Cis-Regulatory Elements in CsPAL

**Figure 3:**
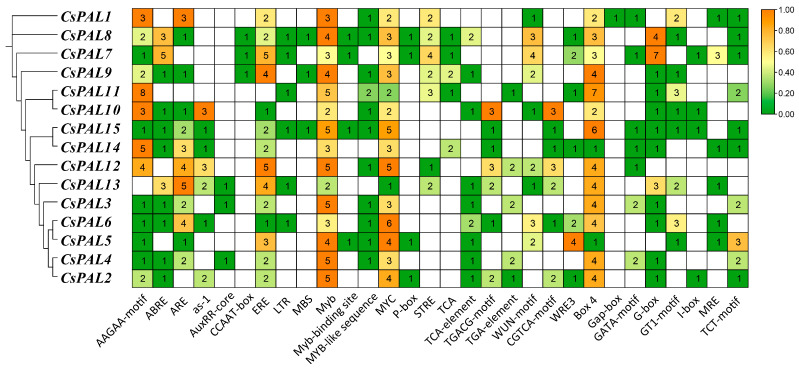
Types and quantities of cis-acting elements in the promoter regions of cucumber *PAL* genes. The numbers in the matrix represent the quantity of cis-acting elements in each CsPAL gene. The higher the number of cis-acting elements, the closer the color is to deep orange; the lower the number, the closer the color is to deep green.

To gain a better understanding of the cis-acting elements present in the promoter regions of the cucumber *PAL* gene family and investigate how the family responds to stressors, we conducted a prediction of the cis-acting elements within the 2000 base pairs upstream of the cucumber *PAL* gene family. The prediction results revealed numerous elements involved in light, hormone, and abiotic stress responses in the cucumber *PAL* gene family (Figure 3). Seven types of hormone response elements and five types of abiotic stress response elements were identified. All *CsPAL* genes contained MYB and MYC elements, totaling 58 and 49, respectively; fourteen *CsPAL* genes contained AAGAA-motif (abscisic acid response element) and ERE (ethylene response element), with totals of 35 and 37, respectively; *CsPAL11* showed a more significant response to the AAGAA-motif; twelve *CsPAL* genes contained ARE elements (anaerobic induction element), totaling 29; eleven *CsPAL* genes contained ABRE elements (abscisic acid response element); nine *CsPAL* genes contained TCA-element (salicylic acid response element) and WUN-motif (wound response element), with totals of 11 and 19, respectively; seven *CsPAL* genes contained as-1 element (salicylic acid response element), STRE (salt stress response element), TGACG-motif (jasmonic acid response element), CGTCA-motif (methyl jasmonate response element), and WRE3 element (wound-induced element), with totals of 13, 16, 13, 13, and 12, respectively; six *CsPAL* genes contained LTR (low temperature response element), totaling six; five *CsPAL* genes contained TCA element (salicylic acid response element) and TGA-element (auxin response element); four *CsPAL* genes contained Myb binding sites and P-box (gibberellin response element), with totals of seven and four, respectively. Only three *CsPAL* genes contained AuxRR-core (auxin response element), CCAAT-box (MYBHv1 binding site), and MBS (MYB binding site induced by drought). The light-responsive elements exhibited the highest diversity and abundance, encompassing several types such as Box4, G-box, GATA-motif, I-box, MRE, TCT-motif, ATC-motif, GT1-motif, Gap-box, etc. It is worth noting that all members of these elements comprise Box4. The findings indicate that the majority of cucumber *CsPAL* family members possess hormone response elements and stress response elements, indicating that the *CsPAL* family may participate in various environmental stress responses through these cis-acting elements.

### 2.3. Chromosomal Location, Tandem Duplication, and Ka/Ks Ratio of CsPAL Genes

**Figure 4:**
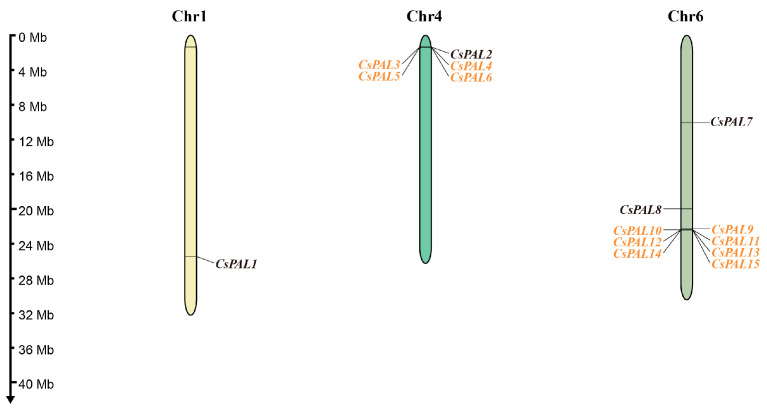
Chromosomal distribution of *CsPAL* in the cucumber genome. The name of each chromosome is presented at the top of the corresponding bar, and the gene names are given on both sides of them. The rules on the left indicate the physical position in megabases (Mb). The genes that underwent tandem repeats are indicated in orange.

Using the annotated cucumber genome data, the locations of *CsPAL* genes were mapped onto the cucumber chromosomes. The results showed an uneven distribution of all genes across three chromosomes: *CsPAL1* is located on chromosome 1 alone, while the rest of the *CsPAL* genes are concentrated on chromosomes 4 and 6 (Figure 4). Studies suggest that tandem duplications tend to amplify genes related to membrane protein functions and those closely associated with biotic and abiotic stresses [28]. To explore the biased retention and selective pressures on the cucumber *PAL* gene family due to tandem duplication during evolution, we performed a duplication analysis on its 15 family members using MCScanX and conducted a Ka/Ks analysis. The duplication analysis revealed that among the 15 cucumber *CsPAL* gene family members, there are nine pairs of genes with tandem duplication relationships: *CsPAL3* and *CsPAL4*, *CsPAL4* and *CsPAL5*, *CsPAL5* and *CsPAL6*, *CsPAL9* and *CsPAL10*, *CsPAL10* and *CsPAL11*, *CsPAL11* and *CsPAL12*, *CsPAL12* and *CsPAL13*, *CsPAL13* and *CsPAL14*, and *CsPAL14* and *CsPAL15* (Table S2). The tandemly repeated *CsPAL* genes may have similar functions. This similarity allows other duplicated *CsPAL* genes to compensate for the function of a mutated or non-functional PAL gene, ensuring the plant’s adaptability to environmental stresses. Ka/Ks analysis showed that the Ka/Ks ratios for all nine gene pairs were less than one, indicating that these gene pairs primarily underwent synonymous substitutions during evolution, maintaining the stability of the protein amino acid sequences, with a relatively low rate of non-synonymous substitutions. This indicates that these gene pairs were under strong negative selective pressure during evolution, maintaining high conservation, and preserving the stability of gene function and structure. Specifically, tandemly duplicated *CsPAL* genes have experienced negative selection pressure, which has helped them retain essential functions. These tandemly duplicated *CsPAL* genes have similar sets of upstream regulatory elements. This similarity suggests that these genes might be co-regulated, maintaining conserved functions despite undergoing duplication. Additionally, the presence of multiple gene copies allows some of these copies to accumulate mutations without compromising the overall biological function, providing new possibilities for evolutionary adaptation.

### 2.4. Protein-Protein Interaction Network Analysis

**Figure 5:**
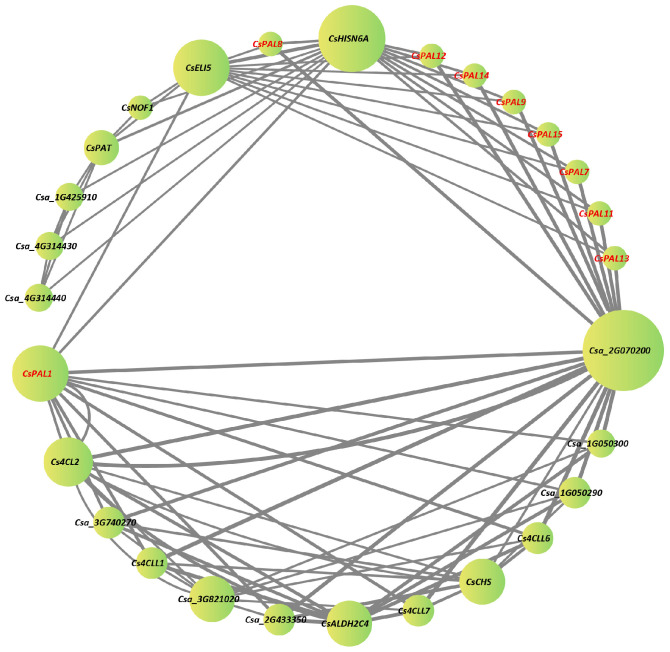
Protein-Protein Interaction network (PPI) of key genes in cucumber. Nodes represent proteins, and black lines indicate interactions between nodes. The size of the node circle is proportional to the number of direct interactions that the gene has with other members in the network; the larger the circle, the more central it is in the network. The thickness of the lines is set according to the R values (correlation values) in the edge file, indicating the strength of the relationship between two nodes. Thicker lines indicate stronger correlations, while thinner lines indicate weaker correlations. The red text represents the proteins of the cucumber PAL family, while the black text represents other proteins that are not part of the PAL family.

Using the online tool STRING, the protein interaction network of the cucumber *PAL* family members was predicted with the cucumber Chinese Long V2 protein database as a reference, identifying interactions among nine family members (Figure 5). No predicted interaction relationships were found among the other 6 cucumber PAL family member proteins. The results obtained from this Protein-Protein Interaction (PPI) analysis suggest that cucumber PAL family members are interconnected through complex multigenic interactions rather than direct interactions. The identification of 28 nodes in the CsPAL protein interaction network highlights the intricate relationships among the proteins involved, with CsPAL1 emerging as a central figure due to its more interactions with other members in the network. This finding indicates that CsPAL1 may play a crucial role in mediating various functions within the PAL family, potentially influencing the phenylpropanoid metabolic pathway, which is vital for plant defense and development. The strong interactions between CsPAL1 and other significant proteins, such as CsELI5, CsHISNA, and Csa_2G070200 (cytochrome P450 cinnamate 4-hydroxylase), are particularly noteworthy. CsELI5 is implicated in ethylene signaling, suggesting that there could be a regulatory link between the PAL family and ethylene response pathways. The interaction with CsHISNA, a gene involved in histidine biosynthesis, points to potential metabolic connections that could affect overall plant growth and stress responses. Additionally, the interaction with cytochrome P450, a key enzyme in the phenylalanine metabolic pathway, reinforces the idea that the PAL family is integral to the biosynthesis of secondary metabolites.

### 2.5. Expression Patterns of CsPAL in Different Tissues

**Figure 6:**
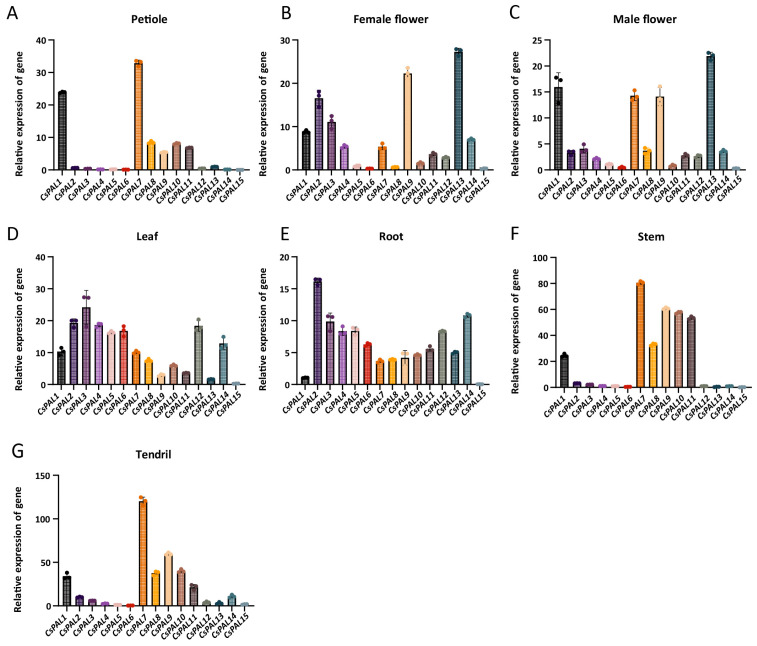
Expression profiles of 15 CsPAL genes in seven different tissues: (**A**) petiole, (**B**) female flower, (**C**) male flower, (**D**) leaf, (**E**) root, (**F**) stem, and (**G**) tendril. Expression levels are presented as the average of three biological replicates (±SD).

To study the expression of *CsPAL* in different tissues, we quantitatively analyzed the expression levels of 15 *CsPAL* genes in female flowers, male flowers, leaves, roots, petioles, tendrils, and stems by qRT-PCR. Figure 6 shows that there are differences in gene expression patterns among different tissues. *CsPAL2*, *CsPAL3*, *CsPAL4*, *CsPAL5*, *CsPAL6*, *CsPAL12*, and *CsPAL14* exhibit higher expression in roots and leaves. *CsPAL9* and *CsPAL13* are expressed at higher levels in both female and male flowers. *CsPAL7* is highly expressed in tendrils, stems, and petioles, while *CsPAL15* has relatively lower expression across all tissues (Figure 6).

### 2.6. Expression Patterns of CsPAL under Abiotic Stress Conditions

Phenylalanine ammonia lyase (*PAL*) plays a crucial role in the plant response to abiotic stresses. We further investigated the response of *CsPAL* to heavy metal stress, drought, and salt stress. Cucumber seedlings were treated with Cu^2+^, Zn^2+^, PEG6000, and NaCl to simulate heavy metal stress, drought stress, and salt stress, respectively, and gene expression was measured. Based on the expression patterns in different tissues (Figure 6), we selected seven *CsPAL* genes that are highly expressed in roots for quantitative analysis: *CsPAL2*, *CsPAL3*, *CsPAL4*, *CsPAL5*, *CsPAL6*, *CsPAL12*, and *CsPAL14*.

**Figure 7:**
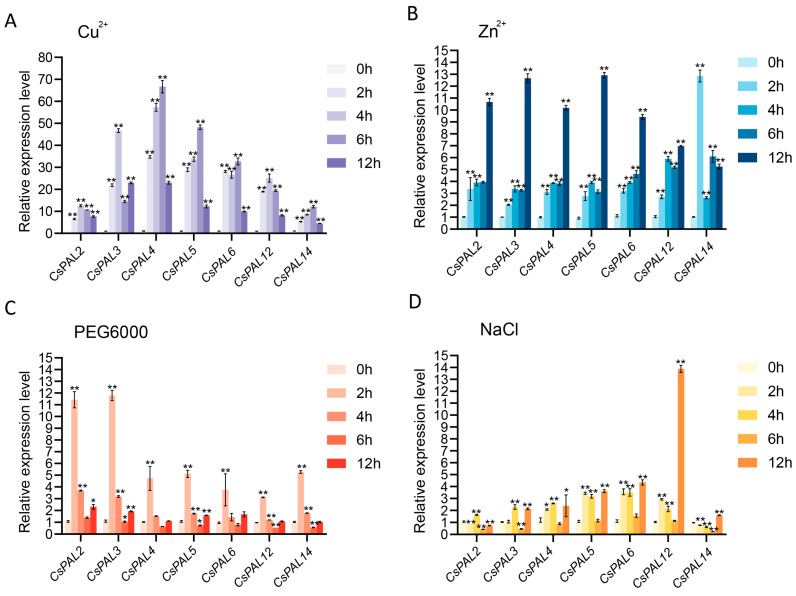
Expression analysis of *CsPAL* genes under various heavy metals, PEG6000, and NaCl. Cu^2+^ (**A**), Zn^2+^ (**B**), drought stress (**C**), and salt stress (**D**) using qRT-PCR. 0 h serves as the control, with samples tested at 2 h, 4 h, 6 h, and 12 h post-treatment. Data are presented as means (±SD) of three biological replicates. Asterisks indicate significant differences by one-way ANOVA: * *p* < 0.05, ** *p* < 0.01.

When compared to untreated controls, the majority of *CsPAL* genes, with the exception of *CsPAL3*, exhibited a pattern of initially increasing and then reducing expression within 12 h after being treated with Cu^2+^. *CsPAL3* exhibited a trend of increasing, then decreasing, and then increasing again (Figure 7A). *CsPAL2*, *CsPAL3*, *CsPAL4*, and *CsPAL6* showed a generally increasing trend within 12 h of Zn^2+^ treatment, reaching their highest expression levels at 12 h; *CsPAL5* and *CsPAL12* showed a trend of increasing, then decreasing, and then increasing again within 12 h of Zn^2+^ treatment, reaching their highest levels at 12 h; *CsPAL14* showed the most complex pattern under Zn^2+^ treatment, with an increasing, then decreasing, then increasing, and again decreasing trend, reaching its peak at 2 h of treatment (Figure 7B). Overall, compared to Zn^2+^ treatment, *CsPAL* showed a more pronounced response to Cu^2+^ treatment. *CsPAL3*, *CsPAL4*, *CsPAL5*, and *CsPAL6* notably responded to both Zn^2+^ and Cu^2+^.

Under treatment with PEG6000, the expression levels of *CsPAL* significantly increased compared to controls, showing the same trend where the most notable change in expression occurred 2 h after treatment. *CsPAL2* and *CsPAL3* showed more than a tenfold increase in expression compared to controls at this time point. After exceeding 2 h of treatment, the expression levels of *CsPAL* generally showed a decreasing trend, with some genes’ expression levels falling below those of the control group. Most genes returned to control levels at 12 h post-treatment. These results indicate that *CsPAL* genes respond rapidly to drought stress, particularly *CsPAL2* and *CsPAL3* (Figure 7C).

Under salt treatment, compared to the control group, the relative expression levels of *CsPAL5* and *CsPAL6* significantly increased by more than threefold, while *CsPAL3* and *CsPAL4* demonstrated a twofold increase. The expression of *CsPAL12* only showed significant differences at 12 h during salt stress treatment. However, the expression changes in *CsPAL2* and *CsPAL14* were minimal. Overall, under salt treatment, the expression levels of *CsPAL* were higher than those of the control group but generally lower than under heavy metal stress and PEG6000 treatment (Figure 7D).

### 2.7. Expression Patterns of CsPAL under Aphid Stress

**Figure 8:**
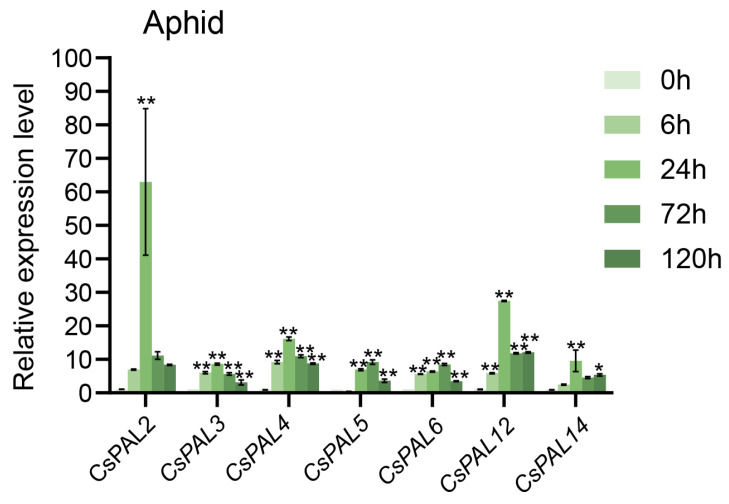
Expression analysis of *CsPAL* genes under aphid stress using qRT-PCR. 0 h is used as the control, with samples tested at 6 h, 24 h, 72 h, and 120 h post-treatment. Data are presented as means (±SD) of three biological replicates. Asterisks indicate significant differences by one-way ANOVA: * *p* < 0.05, ** *p* < 0.01.

Previous studies have shown that *PAL* is involved in plant biotic stress responses. To further investigate the response of *CsPAL* to aphid stress, we selected *CsPAL2*, *CsPAL3*, *CsPAL4*, *CsPAL5*, *CsPAL6*, *CsPAL12*, and *CsPAL14*, which are highly expressed in leaves, and measured their expression changes within 120 h of aphid stress. The results showed that *CsPAL* genes exhibited an initial increase followed by a decrease in expression after aphid treatment. *CsPAL5* and *CsPAL6* reached their peak expression at 72 h under aphid stress, while the other genes showed peak expression after 24 h of aphid stress. After prolonged treatment, the expression levels of all these genes declined but remained significantly higher than the control group. Among them, *CsPAL2* showed the most significant response to aphid stress, with its expression increasing nearly 69 times after 24 h of aphid stress. These results conclude that cucumber *CsPAL* genes exhibit a significant response to aphid stress (Figure 8).

## 3. Discussion

Phenylalanine ammonia lyase (PAL) is a crucial enzyme in the phenylpropanoid system and has been found to have a substantial impact in defending against several environmental stressors, including pathogenic attacks, low temperatures, and heavy metals [29,30,31]. Phylogenetic tree analysis is crucial for studying the evolution of species since it enables the examination of the origins and development of gene families, hence permitting additional investigation into the functionality of genes. This study developed a phylogenetic tree encompassing Arabidopsis, cucumber, melon, tomato, watermelon, grape, maize, wheat, and rice, revealing that the *CsPAL* genes are unequally distributed among three subgroups, which were classified based on the three branches and evolutionary relationships of the phylogenetic tree, suggesting distinct functional roles for these *CsPAL* genes (Figure 1A). Identification of the cucumber *PAL* family genes indicated that cucumber has seven genes distributed over two chromosomes within a single cluster [32]. This contrasts with our findings, where 15 genes identified were distributed across three chromosomes (Figure 4) and located in three subgroups (Figure 1). This discrepancy could be attributed to amendments in the cucumber reference genome and the ongoing progress in research, which consistently uncovers new *PAL* genes. For instance, a study conducted in 2019 on the cucumber *PAL* family uncovered 12 *PAL* family genes [33]. With the advancement of species genomics, processes such as genome recombination, gene duplication, deletion, and mutation may lead to the expansion or contraction of gene families in the future.

Protein sequence alignment of the *CsPAL* family genes in cucumber shows that *CsPAL2* and *CsPAL3* exhibit the most significant sequence differences compared to other genes. Recent studies in cucumbers have indicated that treatment with PGPR (*Pseudomonas* spp.) and Ag-nanoparticles results in increased PAL activity [34]. This is linked to the presence of hormone-responsive cis-acting elements (such as SA and JA) identified in this paper (Figure 3). In cucumbers, *PAL* family genes respond to UV-B irradiation, with transcription levels of *CsPAL4* and *CsPAL10* increasing by 30-fold compared to the control [33]. This response is associated with the light-responsive elements found among the cis-acting elements (Figure 3). Beyond these studies, there are few recent reports specifically focusing on the cucumber *PAL* family genes. Our paper provides a thorough analysis of the cucumber *PAL* family, including gene comparison results combined with gene motifs that reveal *CsPAL* contains a tripeptide active center composed of Ala-Ser-Gly [35]. The absence of this tripeptide active center can lead to reduced or lost PAL enzymatic activity. All 15 genes identified in the *CsPAL* family have this ASG tripeptide active center, therefore possessing PAL activity (Figure 1 and Figure 2). Considering the homology between cucumber *PAL* genes and Arabidopsis genes, it is speculated that genes with high homology have similar functions. *PAL* genes with high homology within the same subfamily are likely to have similar functions. In Arabidopsis, *AtPAL1* and *AtPAL2* regulate flavonoid biosynthesis, while *AtPAL4* is involved in lignin biosynthesis [12,36]. Phylogenetic tree analysis indicates that cucumber PAL family subfamily II (Figure 1A) genes, which are highly homologous to *AtPAL1* and *AtPAL2*, may be involved in the regulation of flavonoid synthesis. Meanwhile, *CsPAL* subfamily III (Figure 1A) genes, which are highly homologous to *AtPAL4*, may be involved in the regulation of lignin synthesis, consistent with the results of expression analysis under stress conditions. The protein interaction network diagram for cucumber *CsPAL* indicates that the *CsPAL* gene family generally interacts with CHS, 4CL, and PAT02 (Figure 5). CHS, as the first rate-limiting enzyme in the flavonoid synthesis pathway, directs the phenylpropane metabolic pathway towards the synthesis of flavonoid compounds. It plays a crucial role in plant growth and development and also serves a significant regulatory function in plant responses to external environmental stresses [37]. Studies have shown that CHS can respond to high-temperature stress and also to MeJA treatment [38], suggesting CHS plays a significant role in plant responses to biotic and abiotic stresses [39]. 4CL is a key enzyme in flavonoid metabolism and also regulates lignin metabolism. Studies in Arabidopsis indicate that *At4CL1*, *At4CL2*, and *At4CL4* are involved in lignin synthesis [40], while Arabidopsis *At4CL3* and rice *Os4CL2* are involved in flavonoid biosynthesis [41,42]. Both lignin and flavonoids are crucial metabolic substances that enable plants to resist stress. Lignin enhances cell wall strength to resist pathogen or insect invasion, while flavonoids, as vital chemical defense substances in plants, mitigate pathogen or pest-induced stress by reducing oxidative reactions in plants [43]. Further research into the interaction mechanisms of PAL and 4CL enzyme genes will facilitate our understanding of the regulatory mechanisms of the phenylalanine metabolic pathway.

The gene evolution tree showed that *CsPALs* and *AtPALs* were closely related, suggesting that cucumber *CsPALs* and *Arabidopsis thaliana AtPALs* had similar functions, and *AtPAL* genes were mostly related to stress responses. Gene structure and cis-acting element analysis also indicated that *CsPAL* genes might be involved in some biotic and abiotic stress responses. To verify the above hypothesis, we treated cucumber with aphid and abiotic stress, and the results also showed that *CsPALs* responded to aphid and abiotic stress. The protein interaction network shows that *CsPALs* interact with some proteins involved in stress resistance, so it is predicted that *CsPALs* may not only respond to stress but also participate in the stress response as resistance genes.

## 4. Materials and Methods

### 4.1. Plant Materials and Growing Conditions

The cucumber variety ‘JinYan 4’ seeds were germinated on filter paper soaked in distilled water for 24 h and then transferred to a growth substrate composed of peat, vermiculite, and perlite (volume ratio 3:1:1). Seedlings at the two-true leaf stage were selected for all the stress treatment experiments. Seedlings were grown in an environment conducive to the growth of both cucumber and aphids, with a light period of 16 h and a dark period of 8 h, at a temperature of 26 °C and 60% humidity.

### 4.2. Aphid and Abiotic Stress Treatment

Aphids (*Aphis gossypii* Glover) were collected from the cucumber fields of Yangzhou University and self-bred for at least six generations before the experiment. Cucumber and aphids were kept in an insect-proof net to ensure a consistent background. For aphid stress treatment, 2–3 instar apterous aphids were starved for 2 h. Then, 40 apterous aphids were evenly inoculated on the first true leaf. Samples were taken at 6 h, 24 h, 72 h, and 120 h after aphid inoculation. Before sampling, aphids were gently removed, and honeydew secretions were wiped off with tissue paper. The central part of each sample leaf was cut out with alcohol-disinfected scissors and used for RNA extraction. For heavy metal stress treatments, 100 µM CuSO_4_ and 500 µM ZnSO_4_ were used. For salinity stress, plant roots were subjected to 150 mM NaCl to simulate salt stress, and 15% PEG6000 to simulate drought stress [44,45]. Control plants were treated with double distilled water. All plants were grown hydroponically, and samples were collected at 2 h, 4 h, 6 h, and 12 h after treatment. Each sample consisted of three biological replicates from three different plants. After cutting, leaf samples were quickly frozen in liquid nitrogen, ground into a fine powder, and stored at −80 °C.

### 4.3. Identification of CsPAL Gene Family Members in Cucumber

The protein sequences of *Arabidopsis thaliana* were obtained from the Arabidopsis Information Resource (TAIR) database (http://www.arabidopsis.org/ (accessed on 10 February 2024)), and PAL homologous sequences in the cucumber whole genome database (http://cucurbitgenomics.org/organism/20 (accessed on 10 February 2024)) were identified through BLAST alignment and filtering (with an E-value threshold set at <10^−10^). The Hidden Markov Model (PF00221) of the PAL protein characteristic domain was downloaded from the Pfam database (https://pfam.xfam.org (accessed on 10 February 2024)) to obtain the conserved domain of PAL. HMMER 3.0 HMM search was used to screen candidate sequences *PAL* genes from the cucumber whole genome database (http://cucurbitgenomics.org/organism/20 (accessed on 10 February 2024)), with an expectation value (e-value) of 1 × 10^−5^. The sequence information of all PAL proteins identified by the above results was submitted to the SMART website (http://smart.embl-heidelberg.de/ (accessed on 10 February 2024)) for cross-validation to confirm the presence of specific domains in the proteins. This validation ensured the reliability of *CsPAL* in cucumber [46].

### 4.4. Physicochemical Properties of CsPAL Family Proteins

The online tool Expasy (http://web.expasy.org/compute_pi/ (accessed on 14 February 2024)) was used to predict the amino acid number, isoelectric point, molecular weight, and hydrophilicity index of *CsPAL* family members.

### 4.5. Analysis of Conserved Motifs, Gene Structure, Cis-Acting Elements, Multiple Sequence Alignment, and Phylogenetic Analysis

From the TAIR database, the Sol Genomics Network, the Rice Genome Database, and the Cucumber Genome Database, full-length amino acid sequences of PAL proteins for the respective species were obtained. Multiple sequence alignments were performed using the MUSCLE function in MEGA11.0 software, and the output data was stored in both MEGA and fasta formats. The resulting fasta files were submitted to GeneDoc to create multiple sequence alignment diagrams; the MEGA files were used to construct phylogenetic trees using the Maximum-Likelihood (ML) method, with a bootstrap value set at 1000. Based on the gene location on the chromosomes, the 15 candidate *PAL* genes in cucumber were named. Using the cucumber genome gff3 file and amino acid sequence information, gene structure analysis of the *CsPAL* family members was performed, with the Gene Structure Display Server 2.0 (http://gsds.gao-lab.org/index.php (accessed on 20 February 2024)) used for visualizing the gene structure analysis. The online tool MEME (http://meme-suite.org/tools/meme (accessed on 20 February 2024)) was employed to analyze the conserved motifs, with a set prediction value of 10 motifs, and TBtools was used for visual motif plotting. Additionally, the nucleotide sequences of 2000 bp upstream of the start codon of each cucumber *PAL* gene were submitted to PlantCARE (https://bioinformatics.psb.ugent.be/webtools/plantcare/html/ (accessed on 20 February 2024)) for predicting cis-acting elements. The obtained results were filtered and categorized based on their regulatory functions.

### 4.6. Chromosomal Localization and Ka/Ks Analysis

Using the distribution information of *CsPAL* family members on chromosomes from the cucumber genome gff3 format file, visual mapping was performed using Mapchart (https://www.omicsclass.com/article/397 (accessed on 22 February 2024)). Ka, Ks, and Ka/Ks of tandemly duplicated genes were calculated using ParaAT (https://ngdc.cncb.ac.cn/tools/paraat (accessed on 22 February 2024)).

### 4.7. Protein-Protein Interaction Prediction

Protein interaction predictions for the *CsPAL* family members were performed using the online tool STRING (https://string-db.org (accessed on 25 February 2024)) with Cucumber Chinese Long V2 as the reference. The maximum number of 1st shell interacting proteins was set to 20, and for the 2nd shell, it was set to 10, with a minimum required interaction confidence of 0.9. Other parameters were set to default values. The protein interaction networks were visualized using Cytoscape (version 3.9.1).

### 4.8. RT-qPCR Expression Analysis

Total RNA from cucumber root and leaf tissues was extracted using the SteadyPure Plant Total RNA Extraction Kit (Hunan Accurate Biotechnology Co., Ltd., Changsha, China). cDNA was synthesized with HiScript III 1st Strand cDNA Synthesis Kit (Vazyme, Nanjing, China). The expression levels of the selected genes were detected by RT-qPCR using the ChamQ SYBR qPCR Master Mix (Vazyme, Nanjing, China) according to the manufacturer’s instructions. Gene-specific primers were designed using the online software Prime5.0 Each treatment was set with three technical replicates. The cucumber actin gene (CsActin) was used as the reference gene to calculate the relative expressions via the 2^−∆∆C^t method. Graphs were plotted and significant differences analyzed using GraphPad Prism v10.0. The sequence of primers used in this paper is shown in Table S3.

## 5. Conclusions

Through bioinformatics analysis of cucumber *CsPAL*, this study identified 15 *CsPAL* genes and conducted a comprehensive analysis of this family, including sequence features, cis-acting elements, tissue specificity, protein interaction network prediction, and expression profiles under abiotic and aphid stresses. The expression profiles showed that *CsPAL2*, *CsPAL3*, *CsPAL4*, *CsPAL5*, *CsPAL6*, *CsPAL12*, and *CsPAL14* all respond to stress to varying degrees, with *CsPAL3*, *CsPAL4*, *CsPAL5*, and *CsPAL6* potentially regulating cucumber’s tolerance to heavy metals and salt stress; *CsPAL2* and *CsPAL3* potentially involved in drought stress; and *CsPAL2* likely playing a significant regulatory role in aphid stress. The findings of this study offer a solid foundation for the selection of *CsPAL* genes in cucumber breeding programs aimed at enhancing resistance to various stressors.

## Data Availability

Data are contained within the article/Supplementary Materials.

## Supplementary Materials

The following supporting information can be downloaded at: https://www.mdpi.com/article/10.3390/plants13182537/s1, Table S1: Physicochemical properties of *CsPAL* family proteins; Table S2: Selective pressure analysis of *CsPAL*; Table S3: Primers used in this study.
