# Supplementary material for: Genome-Wide Characterization and Expression Analysis of CsPALs in Cucumber (Cucumis sativus L.) Reveal Their Potential Roles in Abiotic Stress and Aphid Stress Tolerance
Source: Plants (Basel). 2024 Sep 10;13(18):2537. doi: 10.3390/plants13182537 (PMC11435200; doi:10.3390/plants13182537)
Supplement: Supplementary file 1 [file plants-13-02537-s001.zip › Supplementary Materials.pdf]

Gene Name	Gene ID	Coordinates (5'-3')	ORF (bp)	Protein (aa)	PI	MW (KDa)
CsPAL1	*CsaV3_1G040750*	25961521-25964506	2136	711	5.51	77.87
CsPAL2	*CsaV3_4G002290*	1378889-1379815	927	308	5.33	34.37
CsPAL3	*CsaV3_4G002300*	1379904-1380990	1047	348	8.35	37.43
CsPAL4	*CsaV3_4G002310*	1382724-1385514	2142	713	5.93	77.94
CsPAL5	*CsaV3_4G002320*	1387653-1390118	2142	713	6.00	78.07
CsPAL6	*CsaV3_4G002330*	1392661-1395340	2142	713	6.00	78.06
CsPAL7	*CsaV3_6G014060*	10244020-10248610	2142	713	6.23	77.64
CsPAL8	*CsaV3_6G036550*	20347169-20350749	2142	713	6.26	78.37
CsPAL9	*CsaV3_6G039660*	22800389-22803793	2145	714	6.37	78.03
CsPAL10	*CsaV3_6G039670*	22809979-22812478	2139	712	5.80	77.79
CsPAL11	*CsaV3_6G039680*	22815013-22817701	2127	708	5.76	77.55
CsPAL12	*CsaV3_6G039690*	22819209-22821784	2142	713	6.15	78.05
CsPAL13	*CsaV3_6G039700*	22823980-22826542	2154	717	6.18	78.24
CsPAL14	*CsaV3_6G039710*	22827837-22830468	2118	705	6.12	77.24
CsPAL15	*CsaV3_6G039720*	22831929-22834362	2118	705	5.96	77.17

Table S1: Physicochemical properties of *CsPAL* family proteins

Tandem duplication genes pair	*K*a	*K*s	*K*a/*K*s
*CsPAL*3&*CsPAL4*	0.991579	1.0269	0.965604
*CsPAL*4&*CsPAL5*	0.0138516	0.144335	0.095968
CsPAL5&*CsPAL6*	0.00464371	0.0618532	0.0750762
CsPAL9&*CsPAL10*	0.0560619	1.72707	0.0324607
CsPAL10&*CsPAL11*	0.0352226	0.640454	0.0549964
CsPAL11&*CsPAL12*	0.0410276	1.26918	0.032326
CsPAL12&*CsPAL13*	0.040557	1.13271	0.0358054
*CsPAL*13&*CsPAL*14	0.055194863	0.974349463	0.056647912
*CsPAL*14&*CsPAL*15	0.064559	0.673542	0.09585

Table S2: Selective pressure analysis of *CsPAL*

Primer name	Sequence (5' to 3')
CsActin-F	GCTGGATTCTGGTGATGGTG
CsActin-R	AGCAAGGTCCAAACGGAGAA
CsPAL1-F	AACGGAAACGAAACAGGG
CsPAL1-R	TCGGTAGATTGGGTAAGTGC
CsPAL2-F	CATGCAGTGCTACCTACCCT
CsPAL2-R	TTTCCATTTTCATATGCCAGCC
CsPAL3-F	AGAGCTTGTGCATGAAGGGT
CsPAL3-R	GCTAGCTTTGACACCGGC
CsPAL4-F	CTTGTGCAATACCCATCAGG
CsPAL4-R	CAAAGCCAGTAGTGACACCATA
CsPAL5-F	TCAACTGGGGTTTGGCCG
CsPAL5-R	GTGGCAATGGCAGCAACC

CsPAL6-F	TTGGCCGAGTCTGCAAGG
CsPAL6-R	TGGCACCAAACCCAGTGG
CsPAL7-F	AACGCTGGGATATTTGGG
CsPAL7-R	AGCCTTGGCATTAGGACG
CsPAL8-F	GGAACCCCGTCCAGAAAA
CsPAL8-R	CCTCAGAAAGCTCCACCACA
CsPAL9-F	CTCAACCACAACGTCACTCC
CsPAL9-R	GGCTAACCCTGAACCAACA
CsPAL10-F	CCCAAGTTTGGATTATGGATTC
CsPAL10-R	TTGATTATGTTGCTCGGCAC
CsPAL11-F	TGAGTCCGCTAGAGTCGGT
CsPAL11-R	TGAATGGGGCAAAGTATGAT
CsPAL12-F	ATTGGGGTGTGGCTGCTG
CsPAL12-R	CTTGACAAGGGGCCTCCG
CsPAL13-F	TCCACCAAATCTATTGAGCG
CsPAL13-R	GGCCAAACGAGTGTTATCC
CsPAL14-F	ATACTTTGTCTCGTTCGGCTAC
CsPAL14-R	TGGTTCAAGAGTTTGGTGATG
CsPAL15-F	ACATGCTGGTATTACTTCTGGG
CsPAL15-R	TGCAACTTCTTAGCCGTTTT

Supplementary Table S3: Primers used in this study
